# Stimulation of GLP-1 Receptor Inhibits Methylglyoxal-Induced Mitochondrial Dysfunctions in H9c2 Cardiomyoblasts: Potential Role of Epac/PI3K/Akt Pathway

**DOI:** 10.3389/fphar.2020.00805

**Published:** 2020-05-29

**Authors:** Narawat Nuamnaichati, Supachoke Mangmool, Nipon Chattipakorn, Warisara Parichatikanond

**Affiliations:** ^1^Department of Pharmacology, Faculty of Pharmacy, Mahidol University, Bangkok, Thailand; ^2^Department of Pharmacology, Faculty of Science, Mahidol University, Bangkok, Thailand; ^3^Cardiac Electrophysiology Research and Training Center, Faculty of Medicine, Chiang Mai University, Chiang Mai, Thailand; ^4^Center of Excellence in Cardiac Electrophysiology Research, Chiang Mai University, Chiang Mai, Thailand

**Keywords:** Epac, exendin-4, GLP-1 receptor, methylglyoxal, mitochondrial dysfunction, oxidative stress

## Abstract

Accumulation of methylglyoxal (MG) contributes to oxidative stress, apoptosis, and mitochondrial dysfunction, leading to the development of type 2 diabetes and cardiovascular diseases. Inhibition of mitochondrial abnormalities induced by MG in the heart may improve and delay the progression of heart failure. Although glucagon-like peptide-1 receptor (GLP-1R) agonists have been used as anti-diabetic drugs and GLP-1R has been detected in the heart, the cardioprotective effects of GLP-1R agonists on the inhibition of MG-induced oxidative stress and mitochondrial abnormalities have not been elucidated. Stimulation of GLP-1Rs leads to cAMP elevation and subsequently activates PKA- and/or Epac-dependent signaling pathway. However, the signaling pathway involved in the prevention of MG-induced mitochondrial dysfunctions in the heart has not been clarified so far. In the present study, we demonstrated that stimulation of GLP-1Rs with exendin-4 inhibited MG-induced intracellular and mitochondrial reactive oxygen species (ROS) production and apoptosis in H9c2 cardiomyoblasts. GLP-1R stimulation also improved the alterations of mitochondrial membrane potential (MMP) and expressions of genes related to mitochondrial functions and dynamics induced by MG. In addition, stimulation of GLP-1R exhibits antioxidant and antiapoptotic effects as well as the improvement of mitochondrial functions through cAMP/Epac/PI3K/Akt signaling pathway in H9c2 cells. Our study is the first work demonstrating a novel signaling pathway for cardioprotective effects of GLP-1R agonist on inhibition of oxidative stress and prevention of mitochondrial dysfunction. Thus, GLP-1R agonist represents a potential therapeutic target for inhibition of oxidative stress and modulation of mitochondrial functions in the heart.

## Introduction

Mitochondrial dysfunction is associated with diabetes and insulin resistance in many tissues, including the heart. Abnormalities of heart's functions are mediated through pathologic states of insulin resistance and glucose dysregulation (Coats and Anker, 2000; Boudina et al., 2009). For example, myocardial insulin resistance perturbs the metabolic capability of the heart leading to cardiac contractile dysfunction and contributing to the heart failure (HF) (Belke et al., 2000; Mazumder et al., 2004). In addition, dysregulation of insulin signaling in the cardiomyocytes induces oxidative stress and mitochondrial abnormalities (Boudina et al., 2009; Mangmool et al., 2017). The close relationship between the decompensated HF and various mitochondrial abnormalities has been established in humans and experimental animal models (Palaniyandi et al., 2010). According to unbalanced mitochondrial dynamics and functions, the mitochondrial dysfunctions are characterized into divergent patterns include inefficient energy production, excessive mitochondrial reactive oxygen species (ROS) production, apoptosis, interfered mitochondrial calcium homeostasis, and changes in mitochondrial copies, morphology, and mass (Wang and Wei, 2017).

The major endogenous sources of ROS are mainly located in the mitochondrial matrix and high levels of ROS generated from mitochondria can cause cell injury and damage, including apoptosis. In addition, ROS production was increased in the heart of HF patients (Maack et al., 2003) and in animal model of left ventricular (LV) dysfunction (Fukui et al., 2009). Thus, inhibition and/or prevention of mitochondrial dysfunction might improve and slow the progression of chronic diseases, such as diabetes and heart diseases. Even though current therapies are effective for improvement of HF symptoms and provide an acceptable clinical benefit, the marginal impact on HF outcome has still emerged such as an increase in rate of mortality and hospitalization (Yancy et al., 2013). Despite the importance of mitochondrial abnormality in diabetic heart has been revealed, the underlying mechanisms by which mitochondrial dysfunction occurs in cardiomyocytes and develops in patients with HF are not fully clarified. Although the pathophysiology of HF is complex, mitochondrial abnormality seems to be an important therapeutic target for improving cardiac functions (Zhou and Tian, 2018).

Methylglyoxal (MG) is a precursor of advanced glycation end-products (AGEs) and is a major source of intracellular AGEs. Elevation and accumulation of MG has been found in diabetic patients (Matafome et al., 2013), and MG is involved in many cell and tissue damages. For example, treatment with MG causes β-cell dysfunction and induces type 2 diabetes in rats (Dhar et al., 2011). MG also induced endothelial dysfunction and inflammation in a progression of ventricular dysfunction, leading to the development of diabetic cardiomyopathy in diabetic mice (Vulesevic et al., 2016). In cardiac myocytes, MG induced cell injury and apoptosis (Wang et al., 2010) and oxidative stress (Dhar et al., 2016). Hence, MG contributes to the pathogenesis of diabetes and heart disease. In this study, we used MG as a marker for the induction of oxidative stress and mitochondrial dysfunction. It is crucial to discover the new potential therapeutic strategies to suppress and reverse underlying cardiac dysfunction induced by MG.

Glucagon-like peptide 1 receptor (GLP-1R) agonists (also called GLP-1 analogs) were approved for treatment of type 2 diabetes. GLP-1 and GLP-1R agonists play an essential role on glucose homeostasis. Indeed, GLP-1Rs are expressed in the heart, and previous studies indicated that GLP-1 and its analogs exhibit cardioprotective effects and play an essential role in the regulation of heart functions. For instance, stimulation of GLP-1R exerts antiapoptotic and antioxidant effects in cardiac myocytes (Mangmool et al., 2015). Administration of GLP-1 increased cardiomyocyte survival and reversed cardiac dysfunctions in the hearts after ischemia-reperfusion injury (Ban et al., 2008). Stimulation of GLP-1Rs improved myocardial functions and prevented cardiac remodeling in mouse infarcted myocardium (DeNicola et al., 2014). Although multiple previous studies have revealed cardioprotective effects of GLP-1R agonists, the mechanisms underlying these effects have not been fully elucidated so far. We hypothesized that cardioprotective effect of GLP-1R agonists might be due to the improvement of mitochondrial dysfunction and inhibition of oxidative stress. However, the underlying mechanisms of these cardioprotective effects, including improvement of mitochondrial functions, following GLP-1R stimulation have not completely clarified.

Agonist stimulation of GLP-1Rs resulted in an activation of adenylyl cyclase (AC) activity which catalyzes the production of cyclic adenosine monophosphate (cAMP) in the cells. cAMP binds to and activates its downstream effectors including protein kinase A (PKA) and cAMP-regulated guanine nucleotide exchange factor (Epac). In cardiomyocytes, exendin-4 attenuated hydrogen peroxide (H_2_O_2_)-induced ROS production and inhibited apoptosis through Epac-dependent pathway (Mangmool et al., 2015). In H9c2 cells, treatment with exenatide improved mitochondrial dysfunction induced by hypoxia/reoxygenation (H/R) through GLP-1R/cAMP pathway (Chang et al., 2018). However, the signal transduction by which GLP-1R inhibited MG-induced oxidative stress, apoptosis, and mitochondrial dysfunction in the heart remains unclear. Therefore, we aimed to investigate GLP-1R signaling pathway for the prevention of MG-induced mitochondrial abnormalities, oxidative stress, and apoptosis in H9c2 cells.

## Materials and Methods

### Materials

Exendin-4, exendin fragment 9-39 (Ex-9-39), GLP-1(9-36) amide, protease inhibitor cocktail, and methylglyoxal (MG) were purchased from Sigma-Aldrich (Saint Louis, MO). Forskolin, 2′,5′-dideoxyadenosine (ddA), PKA inhibitor 14–22 amide (PKI), ESI-09, KN-93, Akt inhibitor VIII, isozyme-selective, Akti-1/2 (Akt inhibitor), and LY294002 were purchased from Calbiochem (San Diego, CA). 8-pCPT-2-O-Me-cAMP-AM or Epac-selective cAMP analog acetoxymethyl ester (ESCA-AM) was from Tocris Biosciences (Bristol, UK). Dulbecco's Modified Eagle Medium (DMEM), phosphate buffer saline (PBS), 0.25% trypsin-EDTA solution, fetal bovine serum (FBS), penicillin/streptomycin solution, and other tissue culture reagents were purchased from Gibco (Grand Island, NY).

### Cell Culture

Rat cardiomyoblast H9c2 cells were from the American Type Culture Collection (ATCC CRL-1446). H9c2 cells were seeded and cultured in DMEM supplemented with 10% FBS and 1% (v/v) penicillin-streptomycin solution at 37°C in CO_2_ incubator as described previously (Parichatikanond et al., 2018). After cells grown to 80% confluent, cells were subcultured with 0.25% trypsin-EDTA solution to maintain cells exponential growth stage.

### Cell Viability Assay

Cell viability was determined using a 3-(4,5-dimethylthiazol-2-yl)-2,5-diphenyltetrazolium bromide (MTT) assay. Briefly, H9c2 cells were seeded in 96-well plates (1 × 10^4^ cells/well) in DMEM with 1% FBS and 1% penicillin-streptomycin for overnight. Cells were treated with various concentrations of MG (1 μM to 10 mM, diluted with water) for 24 h. The medium was replaced with MTT solution (1 mg/ml), incubated 4 h, and DMSO was added to dissolve the insoluble formazan product. The optical density (OD) was measured at a wavelength of 570 nm with a Clariostar microplate reader (BMG Labtech). The results were calculated as a percentage of control cell (non-treated cells). All experiments were performed in triplicate parallels and repeated four times.

### Intracellular ROS Measurement

The intracellular ROS production in H9c2 cells was assessed using a fluorescent dye, 2′,7′-dichlorodihydrofluorescein diacetate (H_2_DCF-DA) (Sigma-Aldrich, USA) as described previously (Mangmool et al., 2015). Briefly, cells were seeded (1 × 10^5^ cells/well) in a 12-well plate overnight before treatment with various agents in serum-free medium condition. After treatment, 10 μM DCFH-DA was added to the cells and then incubated in the dark at 37°C in CO_2_ incubator for 30 min. The fluorescence intensity of DCF was quantified by a Clariostar microplate reader (λ_Ex_/λ_Em_: 485/530 nm).

### Mitochondrial ROS Measurement

The mitochondrial ROS was assessed using a MitoSOX red (Invitrogen). Briefly, cells were seeded onto 12-well plate (1 × 10^5^ cells/well) containing gelatin-coated cover slips overnight before treatment with various agents in serum-free medium condition. After treatment, cells were washed with PBS and incubated with 2.5 μM MitoSOX at 37°C for 10 min. After washing, cells then were mounted with Prolong Diamond Antifade Mountant containing 4,6-diamidino-2-phenylindole (DAPI) (Invitrogen) on glass slides. The fluorescent signal was visualized using an IX-81 fluorescence microscope (Olympus) (λ_Ex_/λ_Em_: 510/580 nm). The average fluorescence intensity per cell (at least 100 cells/experiment) for each experimental group was analyzed using ImageJ analysis software (National institutes of Health). The level of corrected total cell fluorescence (CTCF) was calculated using the following formula, with the data from fluorescence microscopy images, CTCF = integrated density − (area of selected cell × mean fluorescence of background readings).

### Terminal dUTP Nick End-labeling Staining ****Assay****

Terminal dUTP nick end-labeling (TUNEL) staining assay was performed on H9c2 cells by using an *in situ* cell death detection kit (Roche Diagnostics) to evaluate cell apoptosis (Mangmool et al., 2015). Briefly, cells were grown on gelatin-coated round coverslips in a 12-well plate (1 × 10^5^ cells/well) overnight before treatment with various agents in serum-free medium condition. After treatment cells were fixed in 4% paraformaldehyde for at least 2 h, washed with PBS, and permeabilized in 0.1% Triton X-100 for 2 min. The cells were then washed with PBS and subjected to the TUNEL reaction at 37°C in the dark for 60 min. After washing, cells were mounted with Prolong Diamond Antifade Mountant containing DAPI (Invitrogen) on glass slides. The fluorescent signal (green color), emitted by fluorescein-labeled dUTP incorporated into fragmented DNA, was visualized by IX-81 fluorescence microscope (Olympus), and analyzed at least 100 cells in each experiment.

### Mitochondrial Membrane Potential Measurement

Tetramethylrhodamine, ethyl ester (TMRE) was selected to monitor the change in the mitochondrial membrane potential (MMP) by using TMRE Assay Kit (Abcam, Canada). Briefly, H9c2 cells were plated in a 12-well plate (1 × 10^5^ cells/well) overnight before treatment with various agents in serum-free medium condition. Cells were subsequently stained with TMRE for 20 min. The fluorescence value of TMRE was observed on a Clariostar microplate reader (λ_Ex_/λ_Em_: 549/575 nm).

### Western Blotting

The protein expression of Akt, Bax, and Bad was evaluated as described previously with a slightly modification (Phosri et al., 2017). After treatment, H9c2 cells were solubilized in Triton X-100 lysis buffer (20 mM Tris pH 7.4, 0.8% Triton X-100, 150 mM NaCl, 2 mM EDTA, 10% glycerol, 100 μM phenylmethylsulfonyl fluoride, and protease inhibitor cocktail). After centrifugation, amount of protein in cell lysates was measured by a protein assay kit (Bio-Rad) and used bovine serum albumin as a standard. Samples were mixed with 4× SDS loading buffer and denatured by heating at 95°C for 5 min. After that, samples were subjected to SDS-PAGE gels and transferred to PVDF membrane (Bio-Rad), and separately immunoblotted with several antibodies such as Akt (Cell Signaling), phospho-Akt (Cell Signaling), Bax (Cell Signaling), Bad (Cell Signaling), and GAPDH (SantaCruz). Immunoblots were visualized with horseradish peroxidase-conjugated secondary antibodies and a SuperSignal chemiluminescent detection system (Thermo Scientific), using GAPDH as a loading control. The density of the band was calculated using ImageJ software.

### mRNA Analysis by Quantitative Real-Time PCR

The extraction of RNA from H9c2 cells was performed by GeneJET RNA Purification Kit (Thermo Scientific). RT-qPCR was performed on an AriaMx real-time PCR system (Agilent) using KAPA SYBR FAST One-step RT-qPCR kits (KAPA Biosystems). Gene specific primers for mitochondrial markers such as cytochrome c oxidase subunit 5a (COX5a), dynamin-related protein 1 (DRP1), mitochondrial calcium uniporter (MCU), peroxisome proliferator-activated receptor gamma coactivator 1alpha (PGC1α), and pro-apoptotic markers (Bax and Bad) were designed as shown in **Supplementary Table 1**. The expression levels of targeted genes were normalized to those of GAPDH, and were calculated according to the comparative cycle threshold (CT) method. The fold increase in mRNA levels of targeted genes was calculated from 2^-ΔΔ^CT (Parichatikanond et al., 2018).

### Statistical Analysis

Data are presented as mean ± SEM. The statistical analysis was determined using Student's t-test and one-way analysis of variance (ANOVA) followed by Tukey's test. A value of *P* < 0.05 was considered to be statistically significant.

## Results

### Exendin-4 Inhibits MG-Induced Oxidative Stress and Mitochondrial Dysfunction in H9c2 Cells

**Figure 1:**
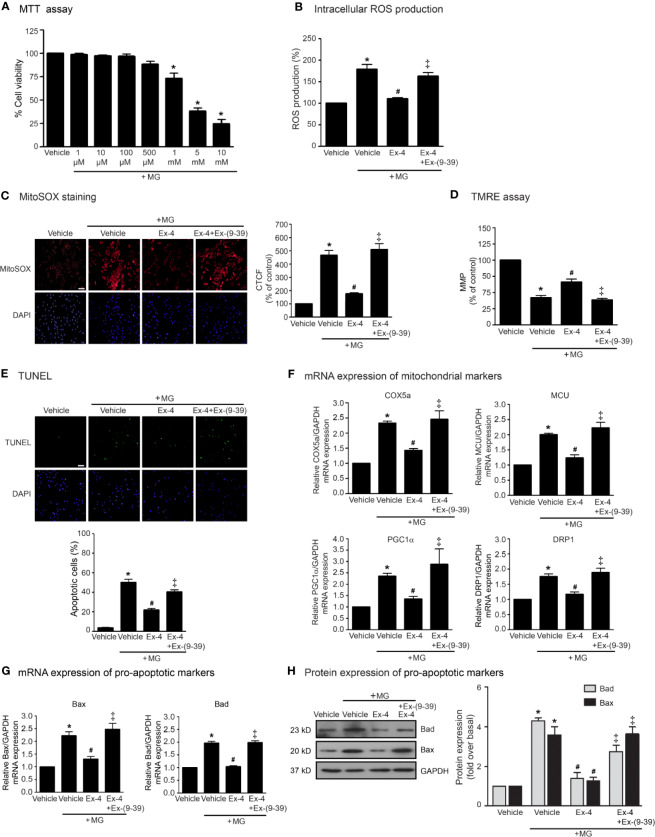
Exendin-4 inhibits MG-induced oxidative stress, apoptosis, and mitochondrial dysfunction in H9c2 cells. **(A)** H9c2 cells were treated with various concentrations of MG for 24 h. Cell viability was quantified and expressed as a percentage of control (non-treated cells). **(B–H)** H9c2 cells were pretreated without or with 100 nM exendin-(9-39) [Ex-(9–39)] for 1 h before treatment with either vehicle (control), or 20 nM exendin-4 (Ex-4) for 3 h. Cells were then incubated with 500 µM methylglyoxal (MG) for the indicated time. **(B)** The intracellular ROS production was quantified and expressed as the percentage relative to vehicle group (control). **(C)** The mitochondrial ROS level was detected by staining with MitoSOX (red), and DAPI (blue) to show nuclei. The fluorescence values were quantified using the corrected total cell fluorescence (CTCF) and expressed as the percentage of control. Scale bar, 10 μm. **(D)** The relative mitochondrial membrane potential (MMP) levels were evaluated and represented as the percentage of control. **(E)** Apoptotic cells were assayed by TUNEL staining (green) and counterstained with DAPI (blue) to show nuclei. The number of apoptotic cells was determined by the percentage of control. Scale bar, 10 μm. **(F, G)** The relative mRNA levels were quantified and expressed as fold increase over control. **(H)** Representative immunoblots are presented for protein expression of pro-apoptotic markers. The relative protein levels were quantified and expressed as fold increase over basal (vehicle) using GAPDH as a loading control. Data are presented as means ± SEM (N = 4). *P < 0.05 vs. vehicle; ^#^*P* < 0.05 vs. MG; ^‡^*P* < 0.05 vs. MG+Ex-4.

We first examined the effects of MG on the cell viability of H9c2 cells using MTT assay and found that MG did not show the cytotoxic effects to H9c2 cells at the concentrations of 1 to 500 µM (**Figure 1A**). We also examined the effects of MG on the induction of oxidative stress and mitochondrial dysfunction. Incubation with MG induced both intracellular and mitochondrial ROS production in H9c2 cardiomyoblasts in a dose-dependent manner (**Supplementary Figure 1**). Based on these results, MG at a concentration of 500 µM was selected for subsequent experiments. We next investigated whether exendin-4 (GLP-1R agonist) inhibits these effects of MG in H9c2 cells. After MG incubation, the productions of intracellular and mitochondrial ROS robustly increased compared to that of vehicle group (**Figures 1B, C**, respectively). Treatment with exendin-4 remarkably attenuated MG-induced intracellular and mitochondrial ROS production. In contrast, pretreatment with exendin-(9–39) (a GLP-1R antagonist) completely antagonized the antioxidant effect of exendin-4 (**Figures 1B, C**, respectively).

The MMP was evaluated by using tetramethylrhodamine, ethyl ester (TMRE) which is a positively-charged fluorophore with red-orange that readily accumulates in active mitochondria. Inactive (depolarized) mitochondria decrease MMP. Alteration/change in the MMP represents an early transition in the apoptotic induction. Incubation with MG robustly reduced MMP, indicating MG contributed to the mitochondrial dysfunction in H9c2 cells (**Figure 1D**). MG-reduced MMP was significantly inhibited by treatment with exendin-4. In contrast, exendin-4 did not improve the alteration MMP induced by MG in the presence of exendin-(9–39) (**Figure 1D**). Furthermore, incubation with MG resulted in an increase of apoptotic cells as detected in the green color assayed by TUNEL staining (**Figure 1E**). Stimulation of GLP-1R with exendin-4 substantially inhibited MG-induced apoptosis in H9c2 cells. Blockade of GLP-1R with exendin-(9–39) antagonized the antiapoptotic actions of exendin-4.

To further assess the protective effects of exendin-4 on mitochondrial dysfunction and apoptosis, we measured the mRNA expressions of many genes involved in mitochondrial functions in H9c2 cells. Incubation of MG potently induced the mRNA expression of genes related to mitochondrial functions (COX5a, MCU, and PGC1α) and mitochondrial fission (DRP1) (**Figure 1F**), and also induced mitochondrial pro-apoptotic mRNA and protein expressions (Bax and Bad) (**Figures 1G, H**, respectively). Stimulation of GLP-1Rs with exendin-4 inhibited MG-induced mRNA expression of functional mitochondrial markers, including COX5a, MCU, PGC1α, and DRP1, highlighting that exendin-4 improves mitochondrial dysfunctions induced by MG (**Figure 1F**). In addition, treatment with exendin-4 restored MG-induced mRNA and protein expressions of Bax and Bad (**Figures 1G, H**, respectively). Blockade of GLP-1R signaling using exendin-(9–39) effectively antagonized these effects of exendin-4. Taken together, these results revealed that exendin-4 possesses the antioxidant and antiapoptotic effects as well as improvement of mitochondrial functions in a GLP-1R-dependent manner.

### Stimulation of GLP-1R Attenuates MG-Induced Mitochondrial Dysfunction in a cAMP-Dependent Pathway

**Figure 2:**
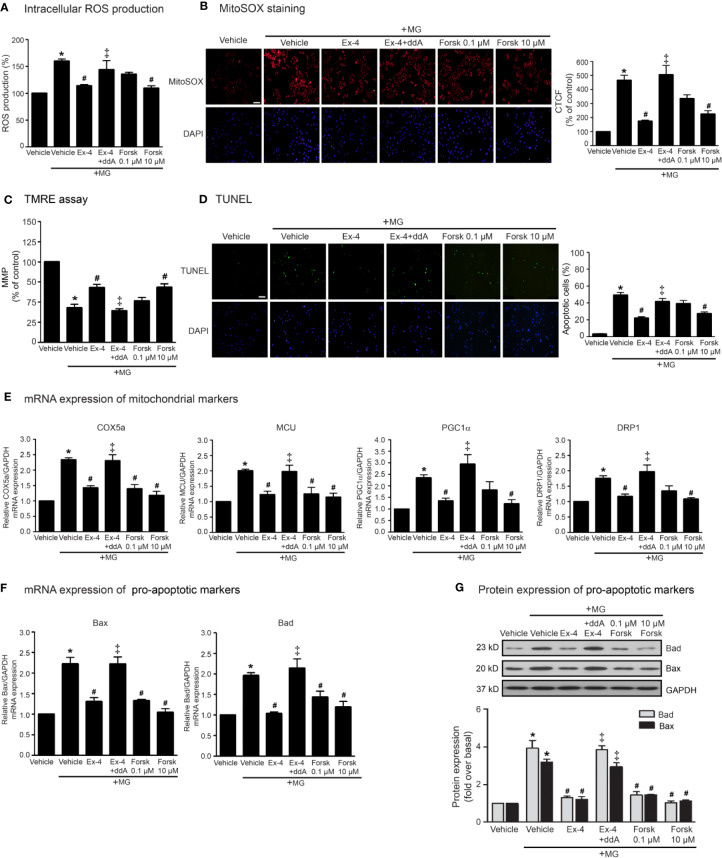
cAMP is required for GLP-1R-mediated inhibition of MG-induced oxidative stress, apoptosis, and mitochondrial dysfunction. **(A–G)** H9c2 cells were pretreated without or with 1 μM dideoxyadenosine (ddA; AC inhibitor) for 1 h before treatment with either vehicle (control), 20 nM exendin-4 (Ex-4), or forskolin (Forsk) for 3 h. Cells were then incubated with methylglyoxal (MG) for the indicated time. **(A)** The intracellular ROS production was quantified and expressed as the percentage relative to vehicle group (control). **(B)** The mitochondrial ROS level was detected by staining with MitoSOX (red), and DAPI (blue) to show nuclei. The fluorescence values were quantified using the corrected total cell fluorescence (CTCF) and expressed as the percentage of control. Scale bar, 10 μm. **(C)** The relative mitochondrial membrane potential (MMP) levels were evaluated and represented as the percentage of control. **(D)** Apoptotic cells were assayed by TUNEL staining (green) and counterstained with DAPI (blue) to show nuclei. The number of apoptotic cells was determined by the percentage of control, Scale bar, 10 μm. **(E, F)** The relative mRNA levels were quantified and expressed as fold increase over control. **(G)** Representative immunoblots are presented for protein expression of pro-apoptotic markers. The relative protein levels were quantified and expressed as fold increase over basal (vehicle) using GAPDH as a loading control. Data are presented as means ± SEM (N = 4). *P < 0.05 vs. vehicle; ^#^*P* < 0.05 vs. MG; ^‡^*P* < 0.05 vs. MG+Ex-4.

According to stimulation of GLP-1R promotes the activation of AC activity through G_αs_ protein, and subsequent elevation of cAMP levels, we determined whether the inhibition of MG-induced mitochondrial dysfunction by exendin-4 is dependent of cAMP. As shown in **Figure 2**, pretreatment with 2′, 5′-dideoxyadenosine (ddA; an AC inhibitor) potently blocked the effects of exendin-4 on prevention of MG-induced intracellular and mitochondrial ROS productions (**Figures 2A, B**, respectively). In addition, treatment with forskolin (AC activator) at a concentration of 10 μM completely inhibited MG-induced intracellular and mitochondrial ROS productions, which had actions similar to those of exendin-4. Furthermore, exendin-4 had no effect on MG-induced the reduction of MMP in the presence of ddA (**Figure 2C**). Interestingly, treatment with forskolin at a low concentration (0.1 μM) tended to inhibit MG-induced MMP reduction and apoptosis, while high concentration (10 μM) of forskolin substantially inhibited these effects (**Figures 2C, D**, respectively), pointing that higher cAMP level confers the protection of mitochondria and prevention of apoptosis induced by MG.

In addition, the inhibitory effects of exendin-4 on MG-induced mRNA expressions of various mitochondrial markers (COX5a, MCU, PGC1α, DRP1), and mRNA and protein expressions of pro-apoptotic markers (Bad and Bax) were blocked by ddA (**Figures 2E–G**). Treatment with 10 μM of forskolin was able to suppress MG-induced mRNA expressions of mitochondrial markers (**Figure 2E**) and mRNA and protein expressions of Bad and Bax (**Figures 2F, G**, respectively). Taken together, these data suggested that stimulation of GLP-1Rs exhibit potent cardioprotective effects through cAMP signaling.

### Epac Is Necessary for GLP-1R-Mediated Inhibition of MG-Induced Oxidative Stress, Mitochondrial Dysfunction, and Apoptosis

**Figure 3:**
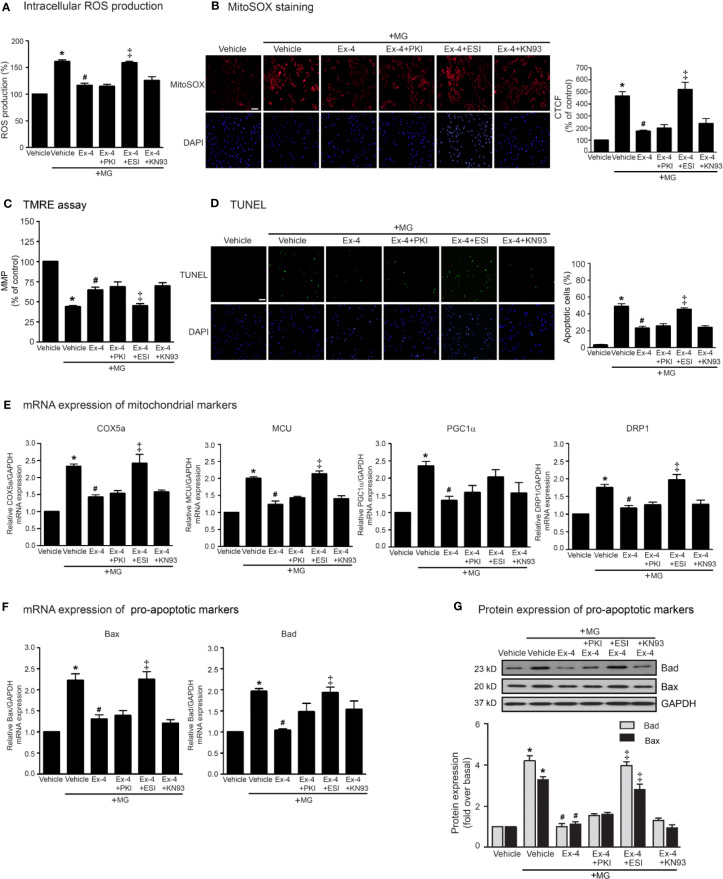
Exendin-4 inhibits MG-induced oxidative stress, apoptosis, and including mitochondrial through Epac-dependent manner. **(A–G)** H9c2 cells were pretreated without or with 10 μM PKI (PKA inhibitor), 10 μM ESI-09 (Epac inhibitor), or 10 μM KN-93 (CaMKII inhibitor) for 1 h before treatment with either vehicle (control) or 20 nM exendin-4 (Ex-4) for 3 h. Cells were then incubated with methylglyoxal (MG) for the indicated time. **(A)** The intracellular ROS production was quantified and expressed as the percentage relative to vehicle group (control). **(B)** The mitochondrial ROS level was detected by staining with MitoSOX (red), and DAPI (blue) to show nuclei. The fluorescence values were quantified using the corrected total cell fluorescence (CTCF) and expressed as the percentage of control. Scale bar, 10 μm. **(C)** The relative mitochondrial membrane potential (MMP) levels were evaluated and represented as the percentage of control. **(D)** Apoptotic cells were assayed by TUNEL staining (green) and counterstained with DAPI (blue) to show nuclei. The number of apoptotic cells was determined by the percentage of control, Scale bar, 10 μm. **(E, F)** The relative mRNA levels were quantified and expressed as fold increase over control. **(G)** Representative immunoblots are presented for protein expression of pro-apoptotic markers. The relative protein levels were quantified and expressed as fold increase over basal (vehicle) using GAPDH as a loading control. Data are presented as means ± SEM (N = 4). *P < 0.05 vs. vehicle; ^#^*P* < 0.05 vs. MG; ^‡^*P* < 0.05 vs. MG+Ex-4.

Following an increase of cAMP levels, cAMP then binds to and stimulates its effectors, such as PKA and Epac. These two effectors regulate many biological functions in the heart. We next investigated the cAMP-dependent signaling pathway of GLP-1R using specific signaling inhibitors (e.g., AC inhibitor, PKA inhibitor, Epac inhibitor, CaMKII inhibitor, PI3K inhibitor, and Akt inhibitor). Treatment with these inhibitors did not interfere with the effects of MG induced oxidative stress and apoptosis in H9c2 cells (**Supplementary Figure 2**). As shown in **Figure 3**, pretreatment with PKI (a specific PKA inhibitor) had no effects on exendin-4-mediated inhibition of MG-induced intracellular and mitochondrial ROS production (**Figures 3A, B**, respectively) and MG-induced MMP reduction (**Figure 3C**). We further investigated the essential role of PKA on exendin-4 affecting on inhibition of MG-induced apoptosis. Inhibition of PKA activity by PKI did not alter the protective effects of exendin-4 on MG-induced apoptosis (**Figure 3D**), MG-induced mRNA expression of COX5a, MCU, PGC1α, and DRP1 (**Figure 3E**), and MG-induced mRNA and protein expressions of Bad and Bax (**Figures 3F, G**, respectively). These results indicated that the protective effects of exendin-4 are independent of PKA.

Epac is one of important downstream effectors of cAMP. We use ESI-09 (a specific Epac inhibitor) to determine the contribution of Epac-dependent signaling pathway on protective effects of GLP-1R agonist. Inhibition of Epac activity using ESI-09 significantly attenuated the protective effects of exendin-4 on MG-induced oxidative stress, apoptosis, and mitochondrial dysfunctions (**Figure 3**). These results demonstrated that Epac is a key regulator for GLP-1R signal transduction.

Ca^2+^/calmodulin kinase II (CaMKII) is one of the downstream mediators of Epac-dependent pathway (Mangmool et al., 2010). We next investigate whether CaMKII is required for GLP-1R–mediated cardioprotective effects. Blockade of CaMKII activity using KN-93 did not block exendin-4–mediated inhibition of MG-induced ROS production, apoptosis, and mitochondrial dysfunctions in H9c2 cells (**Figure 3**), indicating that CaMKII did not play a role on Epac-dependent GLP-1R signaling.

### Stimulation of GLP-1Rs Elicits Cardioprotective Effects Through PI3K-Akt Axis

**Figure 4:**
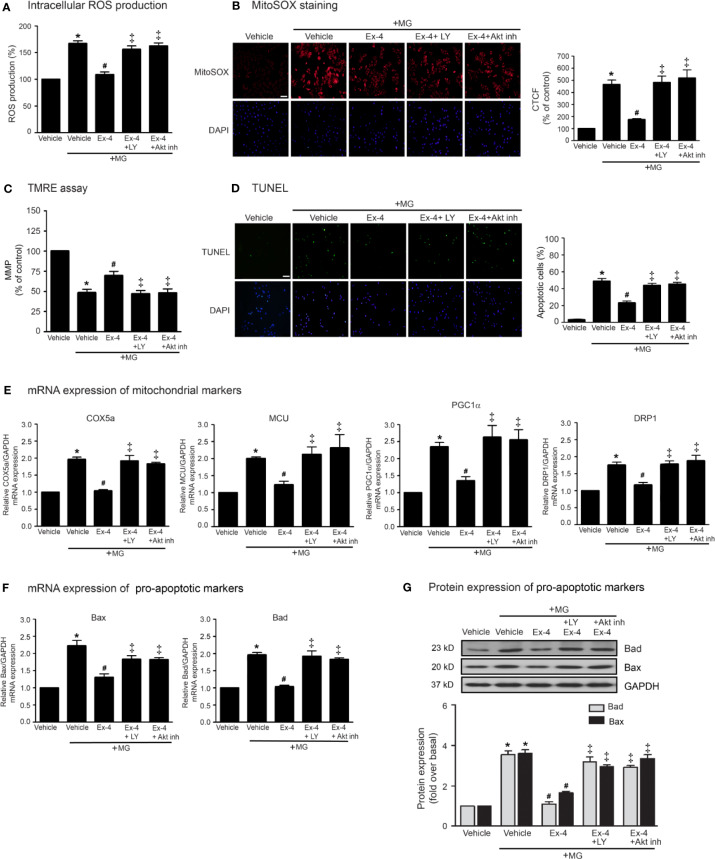
Exendin-4 exhibits protective effects through PI3K-Akt axis in H9c2 cells. **(A–G)** H9c2 cells were pretreated without or with either 10 μM PKI (PKA inhibitor), 10 μM ESI-09 (Epac inhibitor), 10 μM PI3K inhibitor (LY294002) or 1 μM Akti-1/2 (Akt inhibitor; Akt inh) for 1 h. After 1 h, cells were treated with either vehicle (control), or 20 nM exendin-4 (Ex-4) for 3 h, and further induced with methylglyoxal (MG) for the indicated time. **(A)** The intracellular ROS production was quantified and expressed as the percentage relative to vehicle group (control). **(B)** The mitochondrial ROS level was detected by staining with MitoSOX (red), and DAPI (blue) to show nuclei. The fluorescence values were quantified using the corrected total cell fluorescence (CTCF) and expressed as the percentage of control. Scale bar, 10 μm. **(C)** The relative mitochondrial membrane potential (MMP) levels were evaluated and represented as the percentage of control. **(D)** Apoptotic cells were assayed by TUNEL staining (green) and counterstained with DAPI (blue) to show nuclei. The number of apoptotic cells was determined by the percentage of control, Scale bar, 10 μm. **(E, F)** The relative mRNA levels were quantified and expressed as fold increase over control. **(G)** Representative immunoblots are presented for protein expression of pro-apoptotic markers. The relative protein levels were quantified and expressed as fold increase over basal (vehicle) using GAPDH as a loading control. Data are presented as means ± SEM (N = 4). *P < 0.05 vs. vehicle; ^#^*P* < 0.05 vs. MG; ^‡^*P* < 0.05 vs. MG+Ex-4.

Previous studies have been reported that exendin-4 ameliorated β-cells death and enhanced mitochondrial biogenesis through Akt-dependent manner (Fan et al., 2010), and GLP-1 analog protected MG-induced apoptosis *via* EGFR/PI3K/Akt/mTOR pathway in PC12 cells (Kimura et al., 2013). We further investigated whether PI3K and Akt play an important role on GLP-1R–mediated suppression of MG-induced mitochondrial dysfunction in H9c2 cells. We found that blockade of PI3K using LY294002 and blockade of Akt using Akt inhibitor were able to restore exendin-4–mediated inhibitions of MG-induced intracellular and mitochondrial ROS productions (**Figures 4A, B**, respectively), MG-induced TMRE reduction (**Figure 4C**), and MG-induced apoptosis (**Figure 4D**). Furthermore, exendin-4 was able to inhibit MG-induced mRNA expression of COX5a, MCU, PGC1α, and DRP1 (**Figure 4E**), and MG-induced mRNA and protein expressions of Bad and Bax (**Figures 4F, G**, respectively).

**Figure 5:**
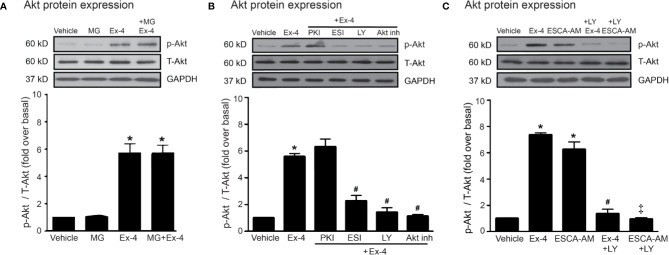
Exendin-4 exhibits protective effects through PI3K-Akt axis in H9c2 cells. **(A)** H9c2 cells were pretreated without or with 500 µM methylglyoxal (MG) for 1 h before treatment with vehicle (control) or 20 nM exendin-4 (Ex-4) for 30 min. **(B, C)** H9c2 cells were pretreated without or with either 10 μM PKI (PKA inhibitor), 10 μM ESI-09 (Epac inhibitor), 10 μM LY294002 (PI3K inhibitor) or 1 μM Akti-1/2 (Akt inhibitor; Akt inh) for 1 h before treatment with vehicle (control), ESCA-AM (Epac activator), or 20 nM Ex-4 for 30 min. **(A–C)** Activation of Akt (phospho-Akt/total-Akt) was quantified and expressed as fold increase over basal (vehicle) using GAPDH as a loading control. Data are presented as means ± SEM (N = 4). *P < 0.05 vs. vehicle; ^#^*P* < 0.05 vs. Ex-4; ^‡^*P* < 0.05 vs. ESCA-AM.

Thus, PI3K and Akt are necessary for GLP-1R/cAMP/Epac-dependent signaling pathway. We next confirmed whether inhibitions of PKA, Epac, and PI3K activities are capable for inhibition of exendin-4–mediated Akt phosphorylation. Following GLP-1R stimulation with exendin-4, phosphorylated Akt (p-Akt) levels robustly increased, whereas treatment with MG had no effect on the phosphorylation of Akt in H9c2 cells (**Figure 5A**). Exendin-4–induced Akt phosphorylation was potently inhibited by ESI-09 (Epac inhibitor) and LY294002 (PI3K inhibitor), but not by PKI (PKA inhibitor) (**Figure 5B**). Furthermore, treatment with 8-pCPT-2-O-Me-cAMP-AM (ESCA-AM; Epac activator) stimulated Akt phosphorylation as shown a similar effect with exendin-4 (**Figure 5C**). In contrast, both exendin-4 and ESCA-AM were unable to activate Akt activity in cells pretreated with LY294002 (**Figure 5C**), pointing that Akt activation by GLP-1R agonist (exendin-4) occurs through PI3K activity. These data demonstrated the novel GLP-1R/cAMP/Epac/PI3K/Akt signaling pathway for cardioprotective effects in H9c2 cells.

### Improvement of Mitochondrial Dysfunction and Inhibition of Oxidative Stress Is Mediated Through Both GLP-1R-Dependent and GLP-1R-Independent Pathways

**Figure 6:**
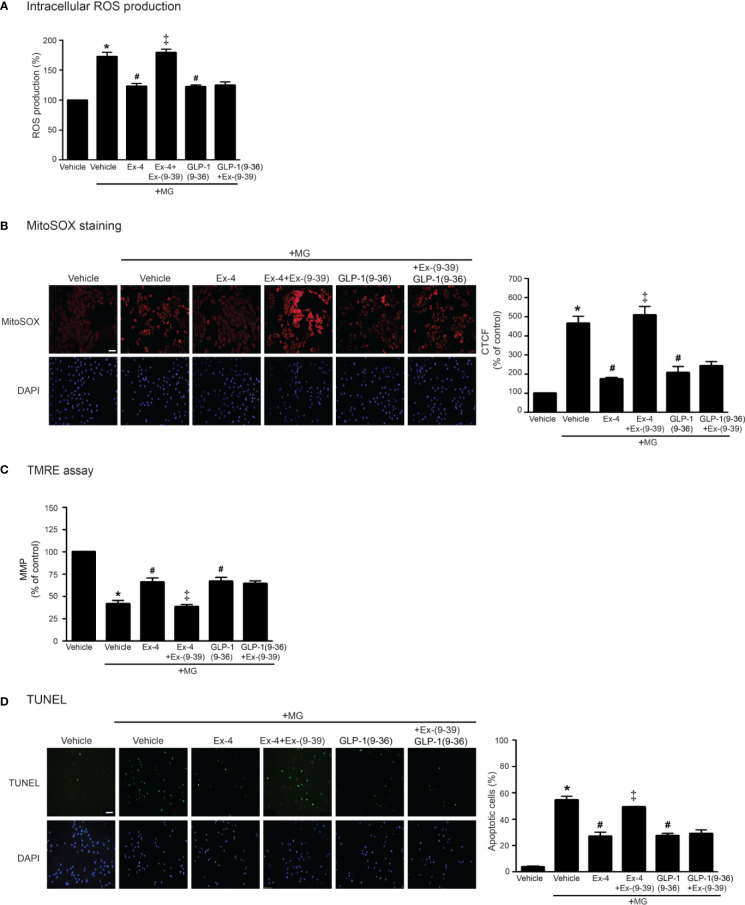
Treatment with either exendin-4 or GLP-1(9–36) prevents MG-induced oxidative stress, apoptosis, and mitochondrial dysfunction in H9c2 cells. **(A–D)** H9c2 cells were pretreated without or with 100 nM Ex-(9–39) (GLP-1R antagonist) for 1 h before treatment with either vehicle (control), exendin-4 (Ex-4) or GLP-1(9–36) (a native GLP-1 metabolite) for 3 h. Cells were then incubated with methylglyoxal (MG) for the indicated time. The intracellular ROS production was quantified and expressed as the percentage relative to vehicle group (control). **(B)** The mitochondrial ROS level was detected by staining with MitoSOX (red), and DAPI (blue) to show nuclei. The fluorescence values were quantified using the corrected total cell fluorescence (CTCF) and expressed as the percentage of control. Scale bar, 10 μm. **(C)** The relative mitochondrial membrane potential (MMP) levels were evaluated and represented as the percentage of control. **(D)** Apoptotic cells were assayed by TUNEL staining (green) and counterstained with DAPI (blue) to show nuclei. The number of apoptotic cells was determined by the percentage of control, Scale bar, 10 μm. Data are presented as means ± SEM (N = 4). *P < 0.05 vs. vehicle; ^#^*P* < 0.05 vs. MG; ^‡^*P* < 0.05 vs. MG+Ex-4.

GLP-1 analog exhibits its effects through GLP-1R (receptor-dependent pathway); however, endogenous GLP-1 is rapidly degraded by dipeptidyl peptidase-4 (DPP-4) to GLP-1(9–36) which has a weak binding affinity to GLP-1R and was previously presumed to have no biological effects (Li et al., 2017). GLP-1(9–36) can activate other signaling pathways that are not involved in GLP-1R/cAMP-dependent pathway (called receptor-independent pathway) (Ban et al., 2008). We further investigated both GLP-1R–dependent and GLP-1R–independent manners for inhibition of MG-induced oxidative stress and mitochondrial dysfunction. As shown in **Figure 6**, treatment with exendin-4 (GLP-1R agonist) remarkably attenuated MG-induced the elevation of both intracellular and mitochondrial ROS levels, which had effects similar to those of GLP-1(9–36) (**Figures 6A, B**, respectively). Interestingly, preincubation with exendin-(9–39) (GLP-1R antagonist) was able to inhibit exendin-4–mediated inhibition of MG-induced oxidative stress and mitochondrial dysfunction, but unable to inhibit GLP-1 (9–36) protective effects (**Figure 6**). Taken together, these results demonstrated that cardioprotective effects against MG-induced mitochondrial dysfunction are mediated through both GLP-1R-dependent and -independent pathways.

## Discussion

In this study, our findings revealed the novel signal transduction of GLP-1R on the inhibition of MG-induced mitochondrial dysfunction in H9c2 cardiomyoblasts. Stimulation of GLP-1Rs exhibits antioxidant and antiapoptotic effects, including improvement of mitochondrial function through cAMP/Epac/PI3K/Akt signaling pathway.

In diabetes, the chronic exposure of high blood glucose levels (hyperglycemia) can stimulate the formation of ROS and the synthesis of toxic by-products of glycolysis, such as MG, leading to the upregulation of AGEs (Giacco and Brownlee, 2010). MG was found to cause mitochondrial injury by disturbance of MMP and suppression of oxidative phosphorylation (OXPHOS) at the respiratory chain complexes contributing to a reduction of ATP production and a raise of ROS levels in mitochondrial matrix (Shangari and O'Brien, 2004; de Arriba et al., 2007). Elevation and accumulation of MG have been implicated in cell injury of many tissues, including the heart (Wang et al., 2010; Dhar et al., 2016; Vulesevic et al., 2016). In the present study, we found that treatment with MG (500 µM) lead to the intracellular and mitochondrial ROS production, apoptosis, and changes of MMP in H9c2 cardiomyoblasts. It should be noted that exposure to MG at the concentration of 500 µM did not cause cytotoxic effects in H9c2 cells. The plasma MG levels in healthy persons are approximately 0.5 µM and can increase to 400 µM in some patients with poorly controlled diabetes (Lapolla et al., 2005). Although the concentration of MG used in our study (500 µM) is higher than the level observed in patients, this high concentration was investigated in various types of cells (Brouwers et al., 2010; Chang et al., 2016). Thus, the effects of MG at various concentrations might be different between whole organisms and living cells as well as in the physiological/pathological environments and cell culture conditions. In our study, the use of MG at a high concentration could represent a model of *in vivo* chronic exposure of MG.

The protective effects of GLP-1 and GLP-1R agonists in many tissues, including the heart, are related with the modulation of mitochondrial functions. For instance, GLP-1 directly targeted mitochondria and preserved mitochondrial functions by suppressing oxidative stress and modulating OXPHOS activity in isolated mouse hepatocytes (Tomas et al., 2011). Exenatide exhibited cardioprotective effects in a model of hypoxia/reoxygenation (H/R) by inhibition of oxidative stress and apoptosis (Chang et al., 2018). Consistent with these previous studies, we demonstrated that exendin-4 exhibits cardioprotective effects by inhibiting MG-induced oxidative stress and apoptosis and restoring the mitochondrial functions of H9c2 cells. Furthermore, stimulation of GLP-1Rs with exendin-4 preserved mitochondrial functions by improving MMP and modulating mitochondrial gene expression.

Mitochondria are dynamic organelles which control the balance of fission and fusion processes. The morphology of mitochondria is controlled by dynamin-related GTPases: DRP1 for fission, and Mfn1/2 and OPA1 for fusion of mitochondrial membrane, respectively (Knott and Bossy-Wetzel, 2008). Mitochondria fission accelerates mitochondrial energetics, ROS generation, and cell proliferation of vascular smooth muscle cells (Salabei and Hill, 2013). Depletion of either DRP1 or mitochondrial fission 1 protein (FIS1) attenuated high glucose-induced inhibition of eNOS activity, likely by reducing mitochondrial ROS production in diabetes (Shenouda et al., 2011). In addition, upregulation of DRP1 contributed to mitochondrial fragmentation and levels of the mitochondrial fission protein DRP1 were increased in a mouse model of type 2 diabetes (*db/db* mice) (Huang et al., 2015). Consistent with these previous studies, here we shown that GLP-1R stimulation by exendin-4 suppressed MG-induced mRNA expressions of DRP1, emphasizing that exendin-4 improves mitochondrial functions in the heart.

PGC1α is a potent transcriptional coactivator that regulates mitochondrial biogenesis and activity in the heart. Dysregulation of PGC1α has been associated with heart failure (Riehle and Abel, 2012). Hypoxia induced PGC1α and mitochondrial biogenesis in cardiac myocytes (Zhu et al., 2010). In the condition of hypoxia and ischemia/reperfusion, hyperphosphorylation of COX subunit I, IV, and V (COX1, COX2, and COX5) inhibited COX activity, resulting in an increase of ROS production (Prabu et al., 2006; Fang et al., 2007). Heart mitochondria isolated from failing heart increased phosphorylation of COX (Rosca et al., 2011). These processes might be an adaptive mechanism for cardiomyocytes to increase ATP output and minimize hypoxic injury to the heart. Mitochondria also regulate calcium homeostasis through matrix Ca^2+^ accumulation by the action of mitochondrial Ca^2+^ uniporter (MCU) (Rizzuto and Pozzan, 2006). Excessive mitochondrial Ca^2+^ accumulation and ROS production resulted in the sustained opening of permeability transition pore complex followed by the mitochondrial permeability transition (MPT), inflammation and cell/tissue damages in ischemic hearts (Griffiths and Halestrap, 1995; Halestrap and Richardson, 2015). In this study, we found that treatment with MG substantially induced the mRNA expression of PGC1α, COX5a, and MCU in H9c2 cells, indicating that changes in mitochondrial functions by MG can be found in the heart. Stimulation of GLP-1R attenuated MG-induced PGC1α, COX5a, and MCU synthesis, pointing the improvement of mitochondrial functions. However, further studies on the precise mechanism underlying improvement of mitochondrial functions of the GLP-1Rs in the heart are still needed.

In mitochondria, a family of Bcl-related proteins, including both anti-apoptotic (Bcl-2, Bcl-xL) and pro-apoptotic (Bad, Bak, Bax) members, involves in initiated apoptosis. Pro-apoptotic Bcl-related proteins regulate the release of cytochrome c from mitochondria (Kale et al., 2018). High glucose level (hyperglycemia) and overproduction of MG are associated with cell injury and apoptosis. Exposure of endothelial cells to either high glucose or MG reduced mRNA expression of the antiapoptotic gene *Bcl-2* and also induced apoptosis (Vulesevic et al., 2016). Moreover, MG induces mitochondrial dysfunction, leading to decrease in ATP/AMP ratio and activation of AMPK. The activated AMPK leads to apoptosis in β-cells (Chang et al., 2016). Interestingly, stimulation of GLP-1R improved mitochondrial function and subsequently increased ATP/AMP ratio, which inhibits these effects of MG in β-cells (Chang et al., 2016). Nonetheless, whether exendin-4 prevents MG-induced apoptosis through suppressing prolonged AMPK activation in the heart remain unknown. Our present study demonstrated that stimulation of GLP-1R with exendin-4 attenuated MG-induced oxidative stress and apoptosis by decreasing ROS production, decreasing a number of apoptotic cells, and reducing the expression of Bax and Bad in cardiomyoblasts. These data emphasize the cardioprotective effects of GLP-1R agonists.

GLP-1 is rapidly degraded by DPP-4 to GLP-1(9–36). The GLP-1(9–36) is a truncated metabolite of GLP-1 which has a weak affinity to GLP-1R and generally thought to be inactive (Drucker et al., 2017). In GLP-1R–overexpressing HEK-293 cells, GLP-1(9–36) has low potency and acts as weak partial GLP-1R agonist (Li et al., 2012). Interestingly, several previous studies have been reported the cardioprotective effects of this GLP-1(9–36) and found that these effects are mediated through GLP-1R-independent pathways. Administration of GLP-1(9–36) in dog model with dilated cardiomyopathy preserved LV function and increased glucose uptake, indicating that GLP-1(9–36) is a biologically active metabolite which is independent of GLP-1R pathway (Nikolaidis et al., 2005). Our present study demonstrated that both exendin-4 (a GLP-1R agonist) and GLP-1(9–36) (a truncated metabolite of GLP-1) provoke cardioprotective effects against MG-induced oxidative stress and mitochondrial dysfunction. Nevertheless, identification and characterization of GLP-1R–dependent and GLP-1R–independent pathways in the heart may lead to a precise understanding of the cardiophysiology of GLP-1 and its analogs.

After exendin-4 binding, GLP-1Rs interact with G_αs_ protein resulting in an elevation of intracellular cAMP levels. cAMP is a major second messenger in the heart where it controls diverse physiological functions such as Ca^2+^ homeostasis, myocardial contractility, and cell death as well as regulation of mitochondrial function (Wang et al., 2016). Elevation of cAMP levels inhibited myocyte apoptosis and reduced mortality in acute myocardial infarction (Takahashi et al., 2006). Administration with liraglutide increased cAMP levels in a GLP-1R-dependent manner in mouse cardiomyocytes (Noyan-Ashraf et al., 2009). Moreover, blockade of AC activity with ddA diminished exendin-4–mediated inhibition of oxidative stress and apoptosis in rat cardiomyocytes (Mangmool et al., 2015). Our present study by using specific activator and inhibitor of AC activity also demonstrated that GLP-1R mediated the improvement of mitochondrial functions through cAMP-dependent manner. cAMP activates PKA and/or Epac to mediate diverse biological effects. Elevation of cAMP levels by roflumilast (a PDE4 inhibitor) induced both PKA-dependent cAMP response element-binding protein (CREB) phosphorylation and Epac-dependent Akt phosphorylation to protect cardiomyocytes against NO-induced apoptosis (Kwak et al., 2008). In addition, stimulation of GLP-1Rs inhibited H_2_O_2_-induced intracellular ROS production *via* Epac-dependent pathway and suppressed H_2_O_2_-induced apoptosis through both PKA- and Epac-dependent signaling pathways in rat cardiac myocytes (Mangmool et al., 2015).

A possible role of Epac in regulating mitochondrial functions has been described and might be reflected by types and localization of Epac proteins in mitochondria (Valsecchi et al., 2013). Stimulation of Epac activity reduced mitochondrial ROS production and prevented cellular injury, apoptosis, and renal failure during reoxygenation after hypoxia (Stokman et al., 2014). Treatment with either a specific Epac activator or forskolin (an AC activator) reduced mitochondrial ROS production in renal epithelial cells (Stokman et al., 2014). In addition, Epac is required for GLP-1R–mediated antioxidation and antiapoptosis in rat cardiac myocytes (Mangmool et al., 2015). Consistent with these previous studies, we showed that blockade of Epac activity antagonized the protective effects of exendin-4 on inhibition of MG-induced mitochondrial abnormalities in H9c2 cells. Data from our study and others suggest the important role for Epac signaling in improving mitochondrial dysfunction and attenuating oxidative stress upon cellular injury/stress.

Activation of PKA inhibits Akt phosphorylation in cortical neurons, whereas activation of Epac induces Akt phosphorylation mediated through Rap activation emphasizing cAMP/Epac/Akt signaling pathway (Nijholt et al., 2008). Induction of Epac activity caused the PI3K-dependent Akt phosphorylation, while activation of PKA inhibited Akt activity in HEK-293 cells (Mei et al., 2002). In addition, Akt phosphorylation was suppressed by either ESI-09 (Epac inhibitor) or LY294002 (PI3K inhibitor) in cardiac fibroblasts (Phosri et al., 2017) and in H9c2 cells (**Figure 5**), suggesting that both PI3K and Akt serve as downstream effectors of Epac-mediated GLP-1R signaling in the heart.

Liraglutide attenuated MG-induced apoptosis in neuroblastoma SH-SY5Y cells through the up-regulations of pro-survival Mcl1 signaling, and Akt and MEK1/2 synthesis (Sharma et al., 2014). Moreover, treatment with GLP-1 inhibited MG-induced apoptosis through EGFR/PI3K/Akt/mTOR/GCLc/redox signaling pathway highlighting its neuroprotective effects in PC12 cells (Kimura et al., 2013). Furthermore, administration of either exendin-4 or GLP-1(9–36) protected cardiac myocytes from I/R injury *via* PI3K and ERK1/2 by increasing the phosphorylations of Akt, ERK1/2, and CREB (Ban et al., 2010). The data from previous studies and our study demonstrated that GLP-1R stimulation leads to activation of Epac, PI3K, and Akt activities in various types of cells and tissues.

## Conclusions

**Figure 7:**
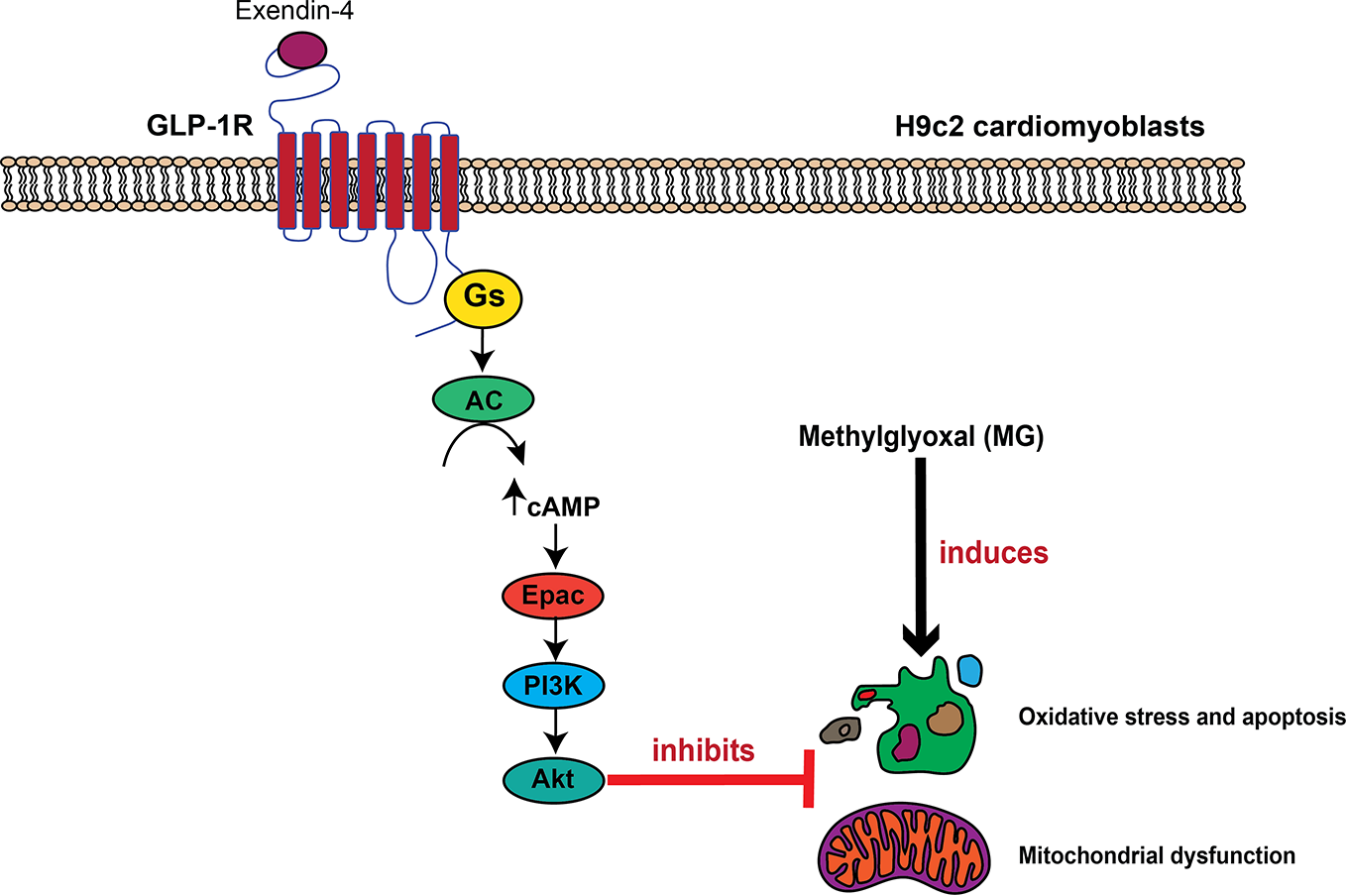
Schematic diagram representing the important role of GLP-1R/cAMP/Epac/PI3K/Akt cascade on inhibition of MG-induced oxidative stress, apoptosis, and mitochondrial dysfunction. In H9c2 cardiomyoblasts, exendin-4 binding to the GLP-1 receptor (GLP-1R) leads to G protein coupling and subsequently causes the stimulation of AC activity through G_αs_ protein, which increases the intracellular cAMP levels. cAMP directly binds to Epac, and subsequently induces Epac/PI3K/Akt signaling pathway, which in turn inhibits oxidative stress, apoptosis, and mitochondrial dysfunction induced by methylglyoxal (MG).

In conclusion, we have identified a novel GLP-1R signaling pathway for inhibition of MG-induced oxidative stress and mitochondrial abnormalities in cardiac cells that mediated through GLP-1R/cAMP/Epac/PI3K/Akt pathway (**Figure 7**). Exendin-4 binding to GLP-1Rs leads to cAMP accumulation through G_αs_ protein and AC activations. Then, cAMP binds to and stimulates Epac activity, leading to activation of PI3K-Akt axis. This present data supports a concept whereby GLP-1R agonists elicit the cardioprotective effects in several *in vivo* studies and in clinical studies.

## Data Availability Statement

All data generated or analyzed during the current study are included in this published article and its **Supplementary Material**.

## Author Contributions

NN carried out the experiments, data, and statistical analysis, and wrote the manuscript. SM performed the experiments, data, and statistical analysis, and reviewed/edited the manuscript. NC contributed to the discussion. WP carried out the experiments and data analysis, participated in the study planning, and wrote the manuscript.

## Funding

This work was supported by the Thailand Research Fund (TRF) through the Royal Golden Jubilee Ph.D. Program, the Office of the Higher Education Commission, Ministry of Education [Grant PHD/0148/2560] (NN), TRF grant MRG6180060 (WP), TRF grant MRG5580037 (SM), and NSTDA Research Chair Grant from the National Science and Technology Development Agency (NC), and Chiang Mai University Center of Excellence Award (NC).

## Conflict of Interest

The authors declare that the research was conducted in the absence of any commercial or financial relationships that could be construed as a potential conflict of interest.

## Abbreviations

AC, adenylyl cyclase; CaMKII, Ca^2+^/calmodulin kinase II; COX5a, cytochrome c oxidase subunit 5a; DAPI, 4,6-diamidino-2-phenylindole; DRP1, dynamin-related protein 1; Epac, exchange protein directly activated by cAMP; GLP-1, glucagon-like peptide-1; MCU, mitochondrial calcium uniporter; MG, methylglyoxal; MMP, mitochondrial membrane potential; PGC1α, peroxisome proliferator-activated receptor gamma coactivator 1alpha; PKA, protein kinase A; ROS, reactive oxygen species; TUNEL, terminal deoxynucleotidyl transferase dUTP nick end labeling.
