# Supplementary material for: Stimulation of GLP-1 Receptor Inhibits Methylglyoxal-Induced Mitochondrial Dysfunctions in H9c2 Cardiomyoblasts: Potential Role of Epac/PI3K/Akt Pathway
Source: Front Pharmacol. 2020 May 29;11:805. doi: 10.3389/fphar.2020.00805 (PMC7274035; doi:10.3389/fphar.2020.00805)
Supplement: Supplementary file 1 [file DataSheet_1.doc]

**Supporting information**

**Supplementary** Table 1. Gene specific primers for RT-qPCR (rat)

| **Gene specific primers** | | **Sequences** |
| --- | --- | --- |
| Cytochrome c oxidase subunit 5a (COX5a) | Sense  Antisense | **5**′**-GACATTGATGCCTGGGAGTT-3**′  **5**′**-CCAAGATGCGAACAGCACTA-3**′ |
| **Dynamin-related protein 1**  (DRP1) | Sense  Antisense | **5**′**-****ATCCAGCTGCCTCAGATTGT-3**′  **5**′**-GTGACCACACCAGTCCCTCT-3**′ |
| Mitochondrial calcium uniporter (MCU) | Sense  Antisense | **5**′**-CGGAGAAGAGGACCACTCTG-3**′  **5**′**-GAAGTAGGTGACCGGTTCCA-3**′ |
| Peroxisome proliferator-activated receptor gamma coactivator 1alpha (PGC1α) | Sense  Antisense | **5**′**-****ATGTGTCGCC TTCTTGCTCT-3**′  **5**′**-CGAGAAAAGGATCTCGAACG-3**′ |
| Bax | Sense  Antisense | **5**′**-CTGCAGAGGATGATTGC TGA-3**′  **5**′**-GATCAGCTCGGGCA CTTTAG-3**′ |
| Bad | Sense  Antisense | **5**′**-** CAGTGATCTGCTCCACATTC**-3**′  **5**′**-** TCCAGCTAGGATGATAGGAC**-3**′ |
| [**Glyceraldehyde 3-phosphate dehydrogenase**](https://www.google.co.th/url?sa=t&rct=j&q=&esrc=s&source=web&cd=1&cad=rja&uact=8&ved=0CB0QFjAAahUKEwjf0oC495PGAhVBIaYKHYWPARI&url=http%3A%2F%2Fen.wikipedia.org%2Fwiki%2FGlyceraldehyde_3-phosphate_dehydrogenase&ei=uvB_Vd-NOMHCmAWFn4aQAQ&usg=AFQjCNHFLovEEmwo3tnUKX-Et3EhrE487Q&sig2=3NYUdBbPtPFVqGFzDqbd1A&bvm=bv.96041959,d.dGY) (GAPDH) | Sense  Antisense | **5**′**-GTGGACCTCATGGCCTACAT-3**′  **5**′**-TGTGAGGGAGATGCTCAGTG-3**′ |

**Supplementary Figure 1. MG induces both intracellular and mitochondrial ROS production in H9c2 cells**

(A-B) Cells were incubated with vehicle (control), 500 μM methylglyoxal (MG), or 1000 μM MG. (A) The intracellular ROS production was quantified and expressed as the percentage relative to vehicle group (control). (B) The mitochondrial ROS level was detected by staining with MitoSOX (red), and DAPI (blue) to show nuclei. The fluorescence values were quantified using the corrected total cell fluorescence (CTCF) and expressed as the percentage of control. Scale bar, 10 μm. **P* < 0.05 vs. vehicle. (N = 3).

**Supplementary Figure 2. Effects of specific signaling inhibitors on MG-induced intracellular and mitochondrial ROS production, and apoptosis in H9c2 cells**

(A-C) H9c2 cells were pretreated with vehicle, 100 nM exendin-(9-39) [Ex-(9-39)], 1 μM dideoxyadenosine (ddA; AC inhibitor), 10 μM PKI (PKA inhibitor), 10 μM ESI-09 (Epac inhibitor), 10 μM KN-93 (CaMKII inhibitor), 10 μM LY294002 (PI3K inhibitor) or 1 μM Akti-1/2 (Akt inhibitor; Akt inh) for 1 h before treatment with 500 μM methylglyoxal (MG) for the indicated time. (A) The intracellular ROS production was quantified and expressed as the percentage relative to vehicle group (control). (B) The mitochondrial ROS level was detected by staining with MitoSOX (red), and DAPI (blue) to show nuclei. The fluorescence values were quantified using the corrected total cell fluorescence (CTCF) and expressed as the percentage of control. Scale bar, 10 μm. (C) Apoptotic cells were assayed by TUNEL staining (green) and counterstained with DAPI (blue) to show nuclei. The number of apoptotic cells was determined by the percentage of control, Scale bar, 10 μm. (N = 3).
