# Supplementary figures and images for: Stimulation of GLP-1 Receptor Inhibits Methylglyoxal-Induced Mitochondrial Dysfunctions in H9c2 Cardiomyoblasts: Potential Role of Epac/PI3K/Akt Pathway
Source: Front Pharmacol. 2020 May 29;11:805. doi: 10.3389/fphar.2020.00805 (PMC7274035; doi:10.3389/fphar.2020.00805)

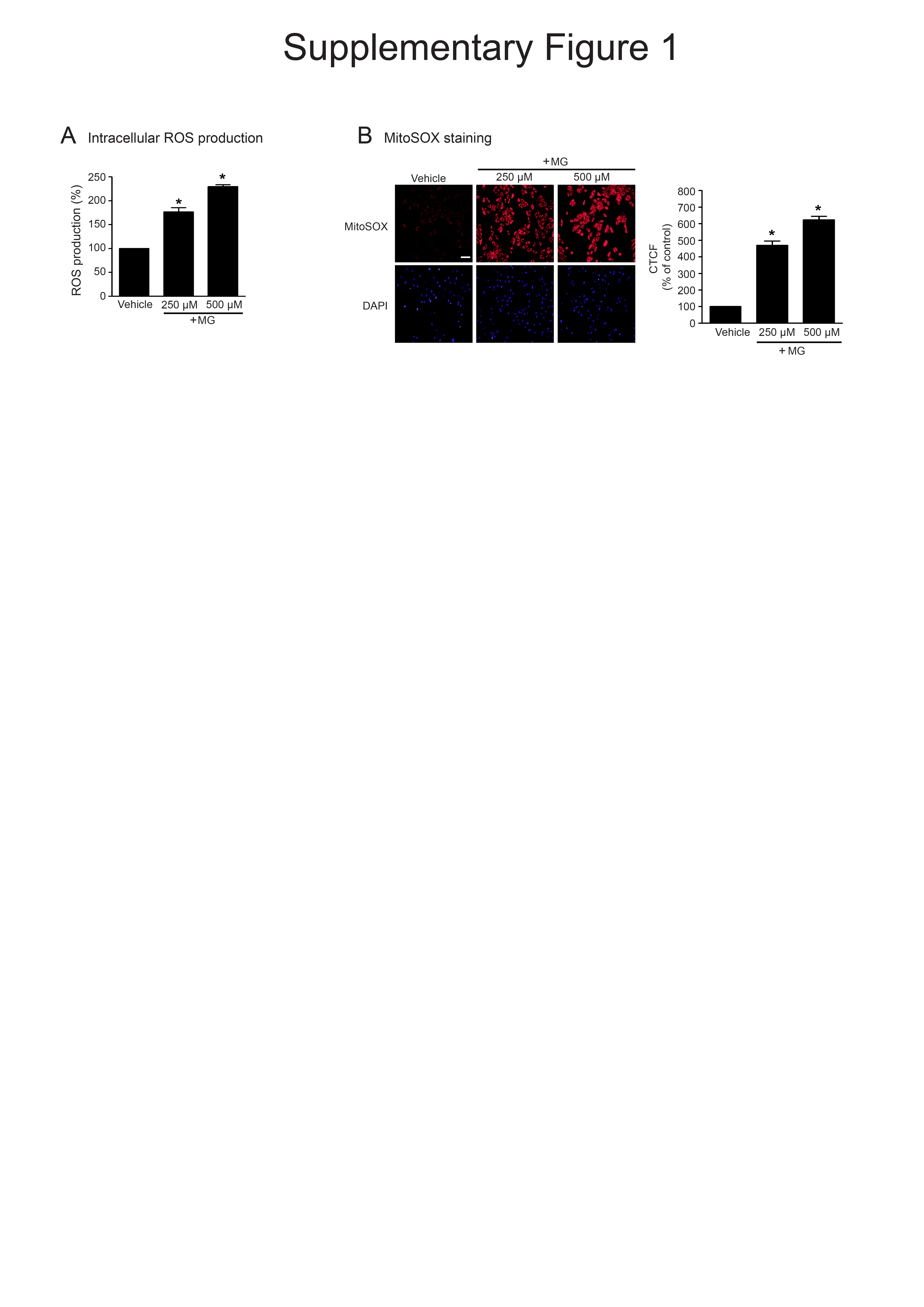

Supplement: Supplementary file 2 [file Image_1.tif]

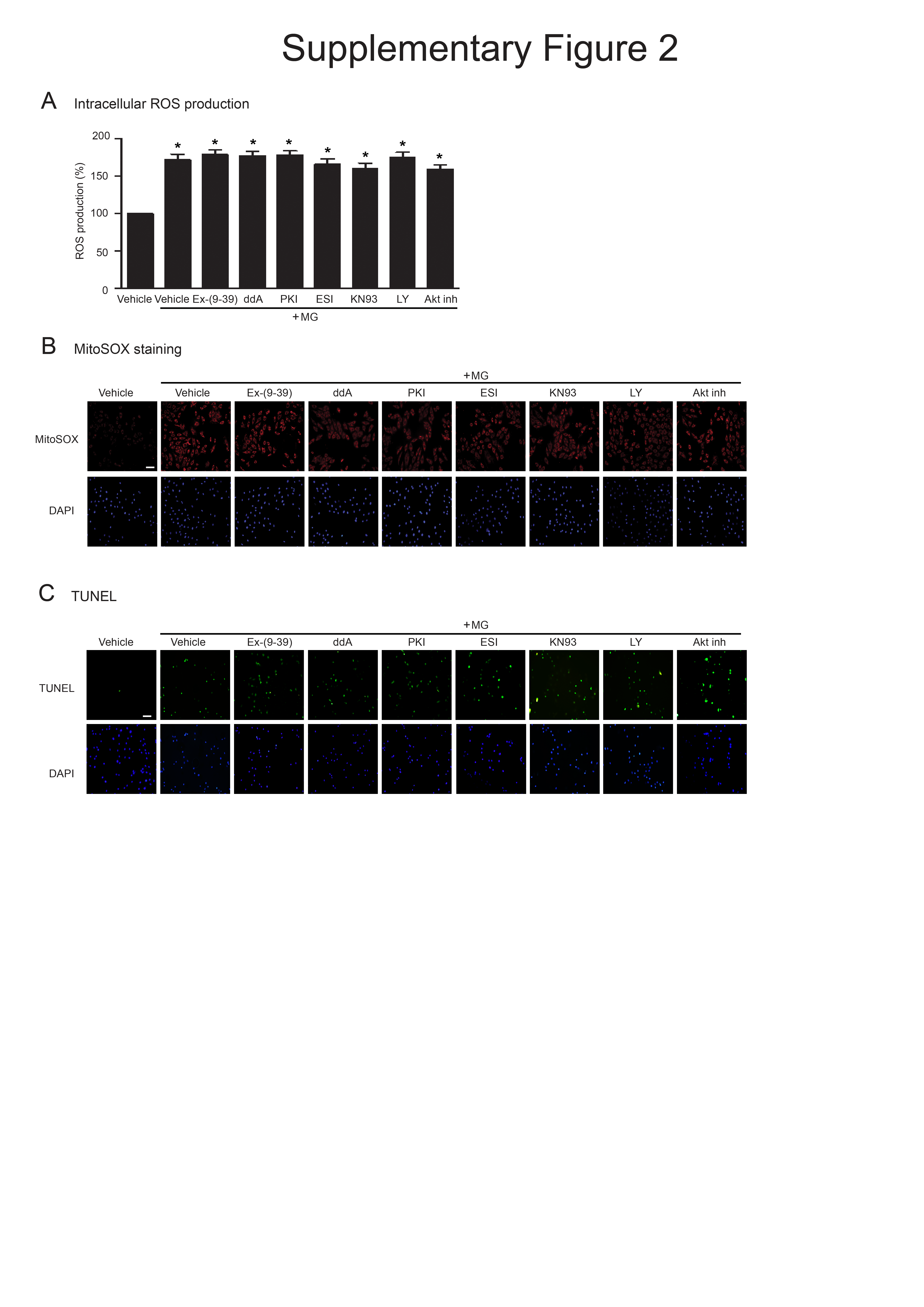

Supplement: Supplementary file 3 [file Image_2.tif]
